# Utilizing the power of plant growth promoting rhizobacteria on reducing mineral fertilizer, improved yield, and nutritional quality of Batavia lettuce in a floating culture

**DOI:** 10.1038/s41598-024-51818-w

**Published:** 2024-01-18

**Authors:** Boran Ikiz, Hayriye Yildiz Dasgan, Nazim S. Gruda

**Affiliations:** 1 Department of Horticulture, Faculty of Agriculture, University of Cukurova, 01330 Adana, Turkey; 2 Institute of Plant Sciences and Resource Conservation, Division of Horticultural Sciences, University of Bonn, 53113 Bonn, Germany

**Keywords:** Plant sciences, Environmental sciences

## Abstract

In soilless cultivation, plants are grown with nutrient solutions prepared with mineral nutrients. Beneficial microorganisms are very important in plant nutrition. However, they are not present in soilless culture systems. In this study we investigated the impact of introducing Plant Growth Promoting Rhizobacteria (PGPR) as an alternative to traditional mineral fertilizer in hydroponic floating lettuce cultivation. By reducing mineral fertilizers at various ratios (20%, 40%, 60%, and 80%), and replacing them with PGPR, we observed remarkable improvements in multiple growth parameters. Applying PGPR led to significant enhancements in plant weight, leaf number, leaf area, leaf dry matter, chlorophyll content, yield, and nutrient uptake in soilles grown lettuce. Combining 80% mineral fertilizers with PGPR demonstrated a lettuce yield that did not significantly differ from the control treatment with 100% mineral fertilizers. Moreover, PGPR application improved the essential mineral concentrations and enhanced human nutritional quality, including higher levels of phenols, flavonoids, vitamin C, and total soluble solids. PGPR has potential as a sustainable substitute for synthetic mineral fertilizers in hydroponic floating lettuce cultivation, leading to environmentally friendly and nutritionally enriched farming.

## Introduction

The utilization of synthetic chemical fertilizers has become indispensable in conventional agriculture in abundance^1^. In addition, the agriculture sector faces a pressing issue due to the increasing imbalanced utilization and extreme costs associated with chemical fertilizers^2^. A potential solution involves optimizing crop management practices, and improving resource use efficiency. Applying "biostimulants and biofertilisers" emerges as an innovative, natural, environmentally friendly, sustainable, cost-effective technology, playing an essential role in addressing these challenges^3,4^. The use of biofertilizers, which contain living microorganisms include beneficial fungi, bacteria and algae, enables the sustainable preservation of soil's physical, chemical, and biological structure, as well as facilitates plants' more efficient utilization of the synthetic mineral fertilizers. They can provide sufficient nutrients to the plants, resulting in high yields^5^. Further, they not only minimize the dependence on chemically synthesized fertilizers, but also directly benefit plants by providing macro and micronutrients and plant growth-promoting hormones^2,6^. In addition, the biofertilizers improve quality, increase crop stress tolerance, enhance soil microbiome, and protect against pathogens^7–9^.

The direct effects of Plant Growth Promoting Rhizobacteria (PGPR) are providing phytonutrients such as biological fixation of nitrogen or solubilized minerals like phosphorus (P), potassium (K), zinc (Zn), iron (Fe), and other essential mineral nutrients^10–12^. This support plant growth and health. The PGPRs can regulate phytohormone levels in plants, including auxins, cytokinins, gibberellins, abscisic acid, and ethylene. By modulating the levels of these phytohormones, PGPRs can influence various aspects of plant growth, such as root and shoot development, flowering, and stress tolerance^6^. The indirect effects of PGPRs are suppressing phytopathogens, and harmful microorganisms through various mechanisms contributing plant protection against diseases as biological control agents^11^. Reported PGPR encompass a diverse array of genera, including but not limited to *Acinetobacter*,* Aeromonas*,* Agrobacterium*,* Allorhizobium*, *Arthrobacter*, *Azoarcus*,* Azorhizobium*,* Azospirillum*,* Azotobacter*,* Bacillus*,* Bradyrhizobium*,* Burkholderia*,* Caulobacter*,* Chromobacterium*,* Delftia*,* Enterobacter*,* Flavobacterium*,* Frankia*,* Gluconacetobacter*,* Klebsiella*,* Mesorhizobium*,* Micrococcus*,* Paenibacillus*,* Pantoea*,* Pseudomonas*,* Rhizobium*,* Serratia*,* Streptomyces*,* Thiobacillus*, among others^11^.

Greenhouses frequently employ soilless culture systems (SCS), primarily to cultivate vegetables. New technologies like indoor and vertical farming have recently expanded beyond the greenhouse. In hydroponics, plants grow in a nutrient solution, while substrate culture involves plant roots within an organic, inorganic, or synthetic solid medium^13,14^. Mineral fertilizers are the sole source of nutrition for plants in conventional soilless cultivation^15,16^. The SCS can improve water use efficiency, especially in closed-loop systems that collect and recycle water for reuse^17^. Unlike in soil-based systems, SCS lack beneficial microorganisms in the root environment, depriving plants of their advantages. To compensate for this, recently, there has been an increasing emphasis on integrating biofertilizers into hydroponic cultivation techniques to reduce reliance on mineral-based fertilizers while simultaneously improving plant nutrition, yield and nutritional quality for human health^9,18,19^.

The use of PGPR in soil-grown lettuce has been explored in several studies^20–22^. Growing leafy green vegetables in SCS, a particular and rigorous system, necessitates substantial production knowledge, experience, technical expertise, and financial investment compared to other greenhouse crops^23^. Lettuce (*Lactuca sativa* L.), a leafy vegetable belonging to the family of the *Asteraceae*, with many different types and cultivars, is one of the prevalent soilless cultivated leafy vegetables. Consumers demand lettuce at all times of the year. It is a cool-season crop that thrives within a temperature range of 7 to 24 °C. Lettuce is commonly consumed as part of salad mixes, and it is nutritious, serving as a rich vitamin C, minerals, and dietary fiber source^24,25^. Due to secondary metabolites such as terpenoids, flavonoids, and phenols, lettuce has been used as a medicinal remedy for various human diseases, including stomach problems, inflammation, pain, and urinary tract infections, since ancient times^26^.

Introducing PGPR suggests that hydroponic lettuce nutrient solutions can benefit from reduced reliance on conventional mineral-synthetic fertilizers. Specific proportions may cut costs while promoting environmental health and reducing carbon footprint^18,19^. In closed-loop hydroponic systems, beneficial microorganisms can maximise the utilisation of nutrients by the plant and help regulate pH and EC in the root environment^27^. The addition of biofertilizers can further improve nutrient availability, fertilizer use efficiency and product quality, which is important in human nutrition^9,19,28^. In Turkey, where this study was conducted, the raw materials of mineral fertilisers are expensive because they are imported. In contrast, PGPR bacteria are locally isolated, purified and produced by biotechnological methods Thus, the use of PGPRs in plant nutrition reduces mineral fertilizer use and production costs.

Numerous studies have documented the expansion of PGPR research into SCS^29^, encompassing efforts in salt stress mitigation^30^, and the exploration of nitrogen dosage^31^. However, our study marks a novel approach by introducing PGPR as a substitute for mineral fertilizers in water-based floating culture. We hypothesize that substituting synthetic mineral fertilizers with PGPR in hydroponic lettuce production can conserve fertilizer without compromising plant development, yield, or product quality. This approach can potentially enhance the sustainability of soilless culture systems. The investigation involved a gradual reduction of synthetic mineral fertilizers by 20%, 40%, 60%, and 80%, with PGPR employed as a substitute for the diminished mineral fertilizers.

## Material and method

### Experimental design, plant material and growth conditions

The experiment was conducted in a greenhouse at 36° 59′ N, 35° 18′ E, and 23 m above sea level during the winter growing season in a Mediterranean climate. The research took place from January to March of 2019. The climatic conditions within the greenhouse, spanning dimensions of 45 m in length and 12 m in width, ranged from 18 to 23 °C during the day to 12–16 °C at night, with a relative humidity of 60–70% and exposure to natural sunlight.

**Figure 1:**
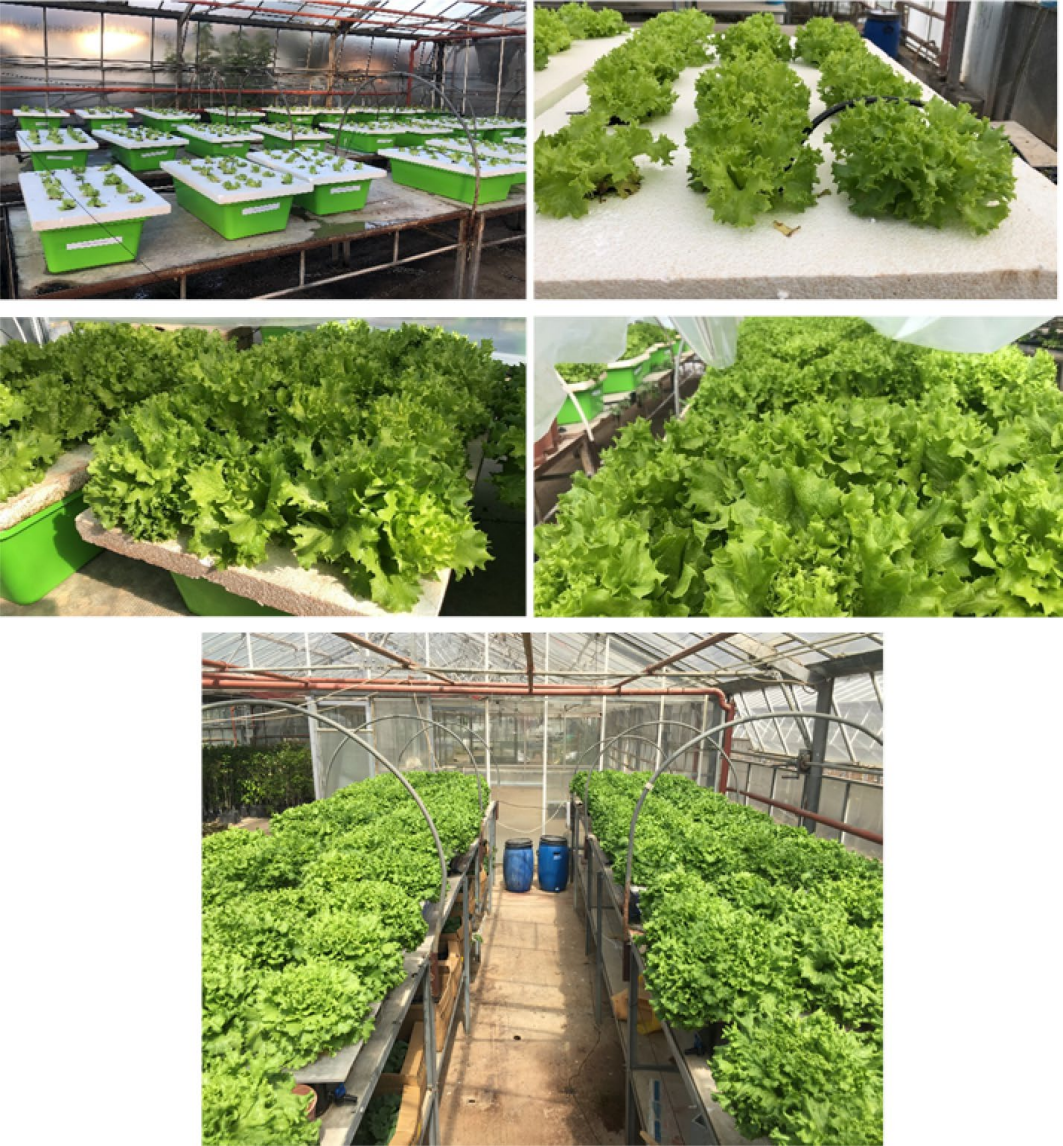
The layout of the experiment in the greenhouse; growing lettuce in floating culture with PGPR using reduced mineral fertilizer.

The Batavia type lettuce (*Lactuca sativa* L. var. *crispa*), cv. ‘Caipira’® from Enza Zaden seed company was used as plant material. A floating culture hydroponic system was established using 50-L cultivation tanks, in which the plant roots were immersed in the aerated nutrient solution. The experiment was laid out in a randomized complete block design with four replicates per treatment and ten plants per replicate, each tank was one replicate (Fig. 1). The plant density was 44.44 plant m^−2^. The plants in the control (100% mineral fertilizers) were supplied with the following nutrient solution^28^ (in mg L^−1^): N (220), P (40), K (312), Ca (210), Mg (65), Fe (5.0), Mn (0.96), Cu (0.30), Zn (0.55), B (0.70) and Mo (0.10). Calcium nitrate, potassium sulphate, mono potassium phosphate, magnesium sulphate, potassium nitrate, Fe–EDDHA, zinc sulphate, boric acid, manganese sulphate, copper sulphate, ammonium molybdate were used for the preparation of nutrient solution^28^. 14 days old lettuce seedlings were transplanted into the hydroponic system. Bacterial inoculation started at the same time as seedling transfer. Lettuce plants were grown for 42 days and harvested. Throughout the cultivation period, the pH and electrical conductivity (EC) of the nutrient solutions were carefully controlled and maintained within the specified ranges of 5.5–6.0 and 1.3–2.2 dSm^−1^, respectively.

### Reduced mineral fertilizer ratios and PGPR treatments

The bacterial biofertilizer utilized in this research is sourced from Next Generation Biotechnology, İstanbul, and is commercially known as Rhizofill®. Rhizofill comprises three distinct pure culture bacteria: *Bacillus subtilis*,* Bacillus megaterium*, and *Pseudomonas fluorescens*. A volume of 50 ml of Rhizofill in a concentration of 1 × 10^9^ colony-forming units per milliliter, was inoculated into the root medium within a 50-L nutrient solution tank (1 ml per liter) at 10-day intervals^9,19,28^. A stepwise reduction of synthetic mineral fertilizers by 20%, employing PGPR as a substitute for the decreased mineral fertilizers:

1. The standard nutrient solution, 100% mineral fertilization as control
2. 100% mineral fertilizer (MN) + PGPR
3. 80% MF
4. 80% MF + PGPR
5. 60% MF
6. 60% MF + PGPR
7. 40% MF
8. 40% MF + PGPR
9. 20% MF
10. 20% MF + PGPR

### Evaluation of plant growth parameters

The weight of harvested lettuce plants was individually measured, and the height of each plant was measured using a ruler. The number of leaves per plant was recorded. The leaf area was determined by leaf area meter (Li-3100, LICOR, Lincoln, NE, USA) and showed as cm^2^ plant^−1^. Information about chlorophyll in the leaves was obtained using a leaf SPAD chlorophyll meter (SPAD-502, Minolta, Osaka, Japan). Lettuce leaves were weighed when fresh (FW), then dried at 65 °C for 24 h and weighed (DW) again to determine the % of dry matter (DW) content (DW = 100xDW/FW)^9^.

### Determination of lettuce yield

Total lettuce yield was expressed as kg m^−2^ at the end of the 42 days of the growing period.

### Determination of micro and macro elements in lettuce leaf

Both macro and micro element analyses were conducted to assess the impact of different treatments on the nutritional status of lettuce plants. For nitrogen (N), phosphorus (P), potassium (K), magnesium (Mg), copper (Cu), and zinc (Zn) analysis, the plant material was subjected to a thorough washing with distilled water. Afterwards, they dried in a forced-air oven set at 65 °C for 48 h. Subsequently, the dried materials were ground using a 40-mesh sieve. The specimens were subjected to dry-ashing in a muffle furnace at a temperature of 550 °C for 6 h. The resulting residue was subsequently solubilized using a solution of 3.3% hydrochloric acid (HCl)^3,32^. The elements K, Ca, Mg, Fe, Mn, Zn and Cu were quantified using an atomic absorption spectrophotometer^3^. The concentrations of N and P in the samples were assessed through the Kjeldahl and Barton methods^3,33^.

### Determination of total phenolics, flavonoids, vitamin C and nitrate in lettuce leaves

To assess total phenolic content, a modified spectrophotometric approach based on Spanos and Wrolstad's procedure was employed^34^. Utilizing a UV-1700 PharmoSpec Shimadzu spectrophotometer (Japan), absorbance at 765 nm was measured, and computations were grounded on a calibration curve established with gallic acid. Analysis of total flavonoids was performed at 415 nm using a spectrophotometer, employing the methodology introduced by Quettier-Deleu et al.^35^. The quantification of total flavonoid compounds was quantified through a calibration curve generated via standard procedures. Vitamin C content was carried out using the adapted approach outlined in Elgailani et al.'s study^36^. A calorimetric method was employed for nitrate analysis, with measurements taken at a wavelength of 410 nm, adhering to the salicylic acid technique as described by Cataldo et al.^37^. Nitrate concentrations were expressed as µg NO_3_-N per unit of fresh weight (ppm)^9^.

### Measurement of EC, pH, and total soluble solids and titrable acidity in lettuce leaves

Lettuce leaf electrical conductivity (EC) and pH were determined at the harvest by using portable pH and EC meters (WTW pH/Cond 3320). Leaf total soluble solids (TSS) were measured with a digital refractometer (Atago PR-101, Tokyo, Japan) and expressed in percentages. Leaf titratable acidity (TA) was measured via potentiometric titration (Mettler Toledo DL22, Milton Freewater, OR, USA), and results were expressed in malic acid percentage^9,19^. This study complies with relevant institutional, national, and international guidelines and legislation.

### Statistical analysis

Data were analyzed using one-way analysis of variance (ANOVA) with the SAS-JMP/13 statistical program. The averages of the treatments were compared with the least significant difference (LSD) test at *p* ≤ 0.05 level.

## Results

The impact of PGPR on lettuce weight was consistently observed across varying levels of reduced mineral fertilizer applications. The effect was most significant when the fertilizer rates were reduced by 80% and 60% (Table 1). The maximum lettuce plant weight recorded was 381 g in the 100% control application. However, there was no statistically significant difference between the 80% MF + PGPR (362 g) and the 100% control group. Employing 80% mineral fertilizer in conjunction with PGPR resulted in lettuce weight being only 5% lower than that achieved with 100% MF. When PGPR was applied, the reduction in lettuce weight was 19.42%, even though the mineral fertilizer was reduced by 60%. The positive impact of PGPR extended beyond weight, manifesting in increased leaf area and leaf dry matter (Table 1). The largest leaf area, measuring 4234 cm^2^, was observed in the 100% MF treatment. The second-largest leaf area of 3959 cm^2^, was attained with 80% MF combined with PGPR. Furthermore, PGPR application resulted in heightened leaf number, increased leaf height, and elevated SPAD-Chlorophyll parameters (Table 2). Regarding using PGPR and a reduced MF by 20 (MF80% + PGPR) exhibited the highest chlorophyll content. Notably, the chlorophyll-SPAD meter contents of all bacterial treatments, except for 20% MF + PGPR, surpassed those of the control treatment.

**Table 1:** Effects of PGPR on plant weight, leaf area and leaf dry matter.

Treatments	Plant weight (g plant^−1^)	Leaf area (cm^2^ plant^−1^)	Leaf dry matter (%)
100% MF	381 a	4234 a	4.54 cd
100% MF + PGPR	336 bc	3915 ab	5.00 bc
%80 MF	279 de	3826 ab	5.19 ab
80% MF + PGPR	362 ab	3959 ab	5.44 ab
60% MF	265 e	3641 ac	4.90 bd
60% MF + PGPR	307 cd	3903 ab	5.24 ab
40% MF	249 e	3364 bc	5.34 ab
40% MF + PGPR	262 e	3586 ac	5.42 ab
20% MF	157 f	2495 d	5.29 ab
20% MF + PGPR	171 f	3086 cd	5.56 a

There is no significant difference between means with the same letter in the same column (*p* < 0.05).

*MF* Mineral fertilizer, *PGPR* Plant growth promoting rhizobacteria.

**Table 2:** Effects of PGPR under the reduced mineral fertilizer conditions on leaf number, leaf height and SPAD-chlorophyll.

Treatments	Leaf number per plant	Plant height (cm)	SPAD-chlorophyll
%100 MF	27.20 a	25.93 bc	24.13 bd
100% MF + PGPR	24.20 bc	26.53 ac	27.86 a
%80 MF	23.60 bc	27.53 ab	27.16 ab
80% MF + PGPR	24.30 bc	28.13 a	29.16 a
60% MF	22,53 c	25,40 c	22.86 cd
60% MF + PGPR	25.00 b	26.60 ac	26.16 ac
40% MF	23.93 bc	25.66 bc	23.90 bd
40% MF + PGPR	24.13 bc	25.86 bc	24.96 bd
20% MF	25.13 ab	21.73 d	21.10 d
20% MF + PGPR	25.06 ab	21.80 d	23.23 cd

There is no significant difference between means with the same letter in the same column (*p* < 0.05).

*MF* Mineral fertilizer, *PGPR* Plant growth promoting rhizobacteria.

### Lettuce yield

**Figure 2:**
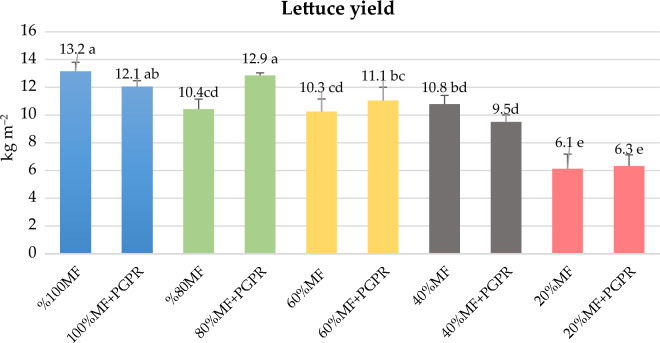
Effects of the PGPR on lettuce yield under the reduced mineral fertilizer levels. *MF* Mineral fertilizer, *PGPR* Plant growth promoting Rhizobacteria. There is no significant difference between means with the same letter in the same histogram (*p* < 0.05)

After a growth period of 42 days, lettuce plants were harvested, and the yield per unit area was calculated in kg m^−2^. A discernible trend emerged wherein the application of PGPR consistently contributed to an augmented lettuce yield compared to their respective controls (Fig. 2). Subsequently, the 80% MF + PGPR application demonstrated a substantial yield of 12.3 kg m^−2^, securing the second position in this assessment. The 100% MF + PGPR application exhibited a lettuce yield of 12.04 kg m^−2^, placing it within the same statistical group as the 80% MF + PGPR application. The 60% MF + PGPR application, which contained 40% less mineral fertilizer, had a higher yield (11.1 kg m^−2^) compared to the yield of the 60% MF application (10.4 kg m^−2^) with a yield difference of 6.7%.

### Mineral element analysis of lettuce

**Figure 3:**
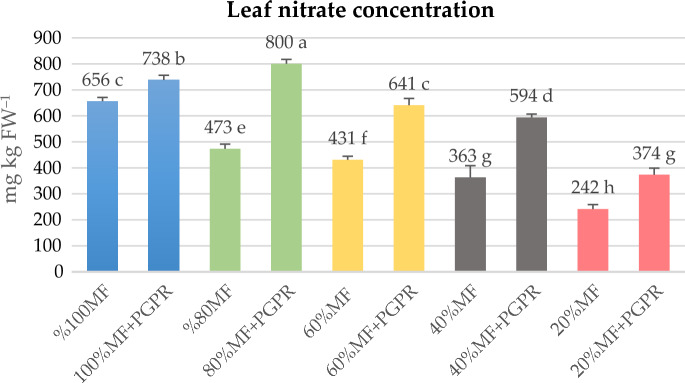
Effects of PGPR on hydroponically grown lettuce nitrate content under the reduced mineral fertilizers. *MF* Mineral fertilizer, *PGPR* Plant growth promoting Rhizobacteria. There is no significant difference between means with the same letter in the same histogram (*p* < 0.05).

Lettuce plants subjected to PGPR treatments exhibited elevated nutrient levels compared to their respective controls. Even under an 80% reduction in mineral fertilizer, nitrogen levels remained higher than its control, suggesting a pronounced influence of PGPR in enhancing nitrogen content. The synergistic application of PGPR up to a 60% reduction in MF was adequate in ensuring sufficient nutrition for key elements such as nitrogen, phosphorus, calcium, magnesium, and potassium. Macro element concentrations were consistently maintained within the nutrient reference ranges established for lettuce plants, as outlined by Campbell^38^. However, it is noteworthy that the PGPR mixture employed in this study did not exhibit the same level of effectiveness as nitrogen, phosphorus, and calcium in providing potassium and magnesium under reduced MF conditions (Table 3). It is plausible that the interaction between the specific lettuce cultivar and the cultivar-PGPR combination might contribute to this observed variability. With regard to micronutrients such as Fe, Mn, Zn and Cu, the effect of PGPR appears to be generally favourable, with microelement concentrations consistently within the nutrient reference ranges outlined by Campbell^38^ for lettuce plants. However, the effectiveness of PGPR in influencing micronutrient levels tends to decrease as the MF level decreases from 100 to 20%. Notably, a significant enhancement in iron uptake was observed with PGPR application. Iron uptake was notably 26% higher in the 80% MF + PGPR treatment compared to its control. The nitrate concentration of the lettuce leaves varied between 241 and 810 mg per kg dry weight in the 20%MF and the 80% MF + PGPR treatments, respectively (Fig. 3). The nitrate concentration of the PGPR-inoculated lettuce was higher in comparison to the non-inoculated one.

**Table 3:** Effects of PGPR on N, P, K, Ca and Mg concentrations of lettuce (g kg^−1^ DW).

Treatment	N	P	K	Ca	Mg
100% MF	35.56 b	11.2 b	70.8 a	8.4 bc	3.5
100% MF + PGPR	40.00 ab	18.4 a	60.6 b	7.6 bc	3.4
%80 MF	25.24 c	8.8 b-d	66.8 a	8.2 bc	3.0
80% MF + PGPR	43.36 a	11.2 b	58.0 b	15.4 a	3.3
60% MF	23.36 c	10.4 bc	32.2 c	9.4 b	3.3
60% MF + PGPR	34.72 b	11.2 b	29.4 c	9.8 b	3.4
40% MF	19.68 cd	4.8 e	30.2 c	5.6 c	3.5
40% MF + PGPR	32.20 b	5.6 e	20.0 d	7.0 bc	3.4
20% MF	13.12 d	6.4 de	8.6 e	3.5 bc	3.6
20% MF + PGPR	20.28 c	7.2 c-e	6.6 e	5.2 c	3.2

There is no significant difference between means with the same letter in the same column (*p* < 0.05).

*MF* Mineral fertilizer, *PGPR* Plant growth promoting rhizobacteria.

### Nutritional quality of lettuce; antioxidants, TSS, pH and EC

**Figure 4:**
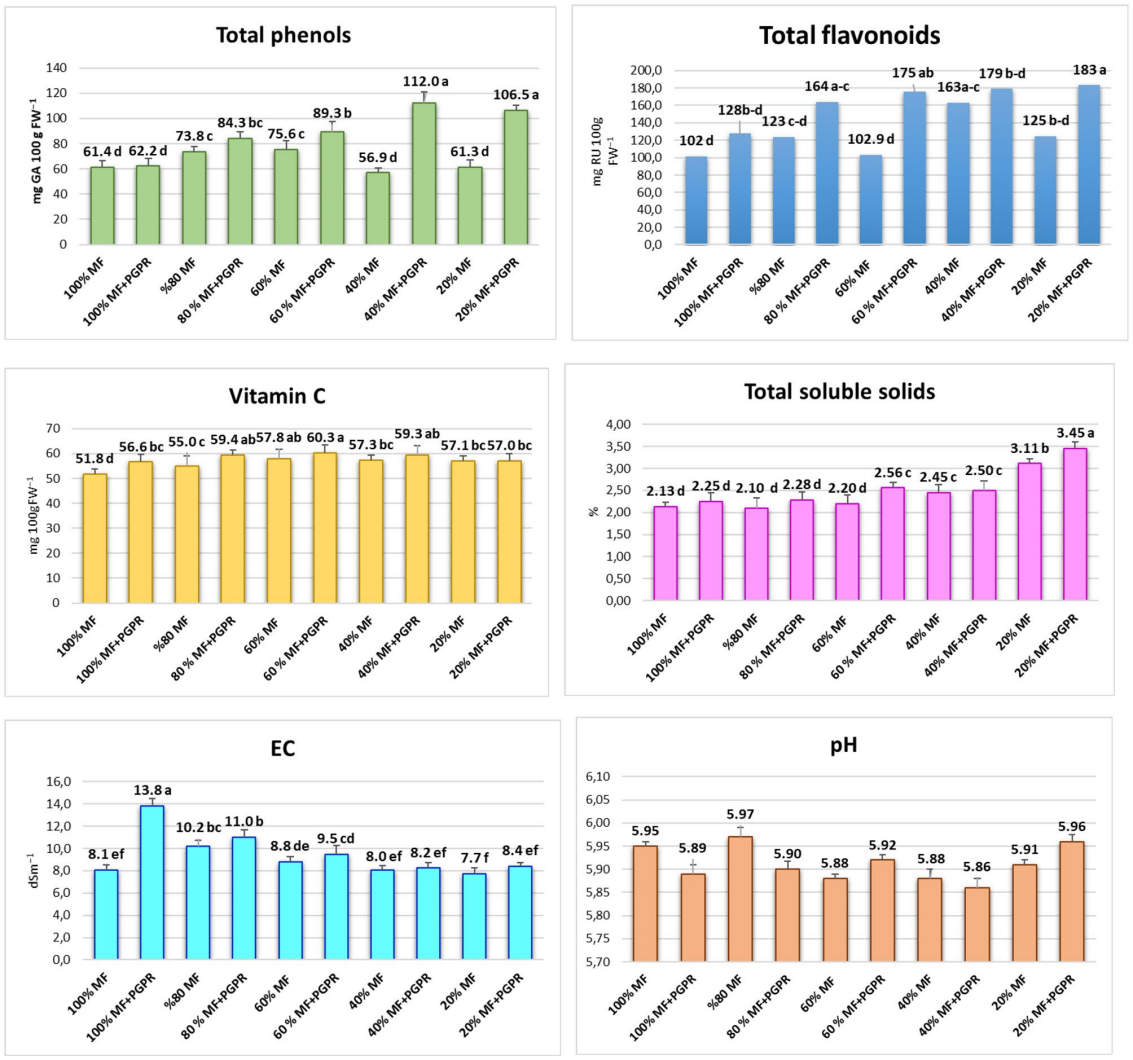
Effects of PGPR on total phenols, total flavonoids, vitamin C, TSS, EC and pH of lettuce. *MF* Mineral fertilizer, *PGPR* Plant growth promoting Rhizobacteria. There is no significant difference between means with the same letter in the same histogram (*p* < 0.05).

The application of PGPR yielded a discernible increase in phenolic compounds when mineral fertilizer was reduced. The phenols in the 40% and 20% MF treatments were highest, as 112.0 and 106.5 mg GA 100 g FW^−1^ (Fig. 4). A parallel trend was observed in flavonoid production, with PGPR-inoculated lettuce demonstrating a capacity for heightened flavonoid production under conditions of reduced mineral fertilizer. The amount of vitamin C was less associated with decreased mineral fertilizer and PGPR application. Vitamin C ranged from 51.8 mg to 60.3 100gFW^−1^, and the highest was obtained by applying 60%MF + PGPR. The PGPR increased the total soluble solids in lettuce leaves. This effect was more significant, especially for decreased mineral nutrition levels (Table 2). The observed increase in electrical conductivity of the lettuce juice following PGPR application aligns with expectations, given the general role of PGPR in enhancing the uptake of mineral nutrients. The pH of the lettuce juice, ranging between 5.89 and 5.95, remained largely unaffected by bacterial inoculation, indicating a robust stability in the acidic profile.

## Discussion

Inoculation of lettuce plants with PGPRs has been shown to promote overall growth and development, as evidenced by advances in key plant growth parameters such as shoot weight, leaf area, plant height, leaf number and dry matter (Tables 1 and 2)^10,20–22^. The symbiotic relationship between bacteria and plant roots reveals a dynamic interplay. In particular, demonstrates the ability to produce phytohormones, particularly indole-3-acetic acid (IAA), which is recognised for its pivotal role in promoting plant vegetative development^22,39^. Furthermore, the beneficial influence of PGPR extends to crucial processes such as nitrogen fixation from the atmosphere and solubilisation of phosphate and potassium in the nutrient solution^10,21,39,40^. These mechanisms contribute synergistically to the overall growth of lettuce plants. In support of this, Acurio Vásconez et al.^10^ reported a significant increase in various growth indicators, including plant height, plant dry matter, plant thickness, root weight and root dry matter, when bacterial strains were inoculated into lettuce plants compared to non-inoculated controls. This noticeable positive effect highlights the proactive role of PGPR in enhancing plant nutrition, promoting higher root growth and ultimately leading to an overall improvement in the growth and development of lettuce plants^20,29^.

By a 100% mineral fertilizer application rate, the efficacy of PGPR was reduced, revealing a nuanced interaction influenced by high nutrient concentrations. The complex dynamics at play suggest that abundant nutrients can inhibit the efficacy of biologically active PGPR. This observation is consistent with the findings of Aini et al.^29^, who state that the combination of PGPR with a reduced dose of recommended synthetic fertilizer results in a better inoculation efficiency. Furthermore, Reid et al.^41^, report that these fertilizers can induce fluctuations in the abundance of PGPR. This finding highlights the sensitivity of the rhizobacterial community to the application of inorganic fertilisers, potentially affecting the overall effectiveness of PGPR. Consequently, a comprehensive understanding of these dynamics becomes imperative for designing cultivation practices that harness the synergistic potential of PGPR in the context of different fertilizer regimes.

In the reduced mineral fertilizer applications, the increase in lettuce yield observed in the presence of PGPR (Fig. 2), is explained by a complex interplay of factors. Firstly, the improvement in plant nutrition is due to the ability of PGPR to increase the solubility, uptake and bioavailability of essential minerals, including phosphorus, potassium, zinc and iron. In addition, the ability to biologically fixate atmospheric nitrogen further contributes to improved nutrient acquisition^10,11,39^. Secondly, the yield-boosting effect of PGPR is closely linked to its ability to stimulate the production of phytohormones such as auxins, cytokinins, and gibberellins. These phytohormones are pivotal in enhancing overall plant growth and development^11,39,40,42^. Thirdly, the effect of PGPRs extends to stimulating the production and excretion of various compounds. These include siderophores, volatile organic compounds and a range of hydrolytic enzymes, including cellulases, pectinases, proteases, catalases and chitinases^43^. This diverse range of compounds act as effective bio-protectants, conferring resistance to various phytopathogens^11,39,42^. The yield data of the presented study is being accordance with findings from the literature. According to Dasgan et al.^9^, PGPR increased basil leaf yield by 18.94% compared to the treatment with 50% mineral fertilizer. Rostaminia et al.^20^ documented a 10–20% rise in lettuce yield by utilizing various *Pseudomonas* bacterial species. The observed increase in lettuce weight and yield can be attributed to the bacteria hydrolysis of ACC (1-aminocyclopropane-1-carboxylic acid). This enzymatic activity reduces ethylene levels around the plant roots, consequently promoting an augmentation in both root and shoot weights. Tahiri et al.^21^ reported a 58.7% higher lettuce yield in PGPR-inoculated plants compared to non-inoculated plants. Vetrano et al.^22^ reported a 14–25% increase in lettuce yield due to bacterial inoculum and fertigation management. The increased leaf area, leaf number and chlorophyll content and the increased availability and uptake of nutrients lead to increased photosynthesis and the accumulation of higher biomass and dry matter of the lettuce plant (Tables 1, 2, 3, 4 and Fig. 2).

**Table 4:** Effects of PGPR on Fe, Mn, Zn and Cu concentrations of lettuce (mg kg^−1^ DW).

Treatment	Fe	Mn	Zn	Cu
%100 Control	251 b	64 a	213 a	5.03 b
100% MN + PGPR	248 b	59 b	198 b	4.96 b
80% MN	211 c	51 c	169 c	4.93 b
80% MN + PGPR	266 a	59 b	201 b	5.33 a
60% MN	140 d	36 d	112 d	3.08 c
60% MN + PGPR	154 d	33 d	123 d	2.80 c
40% MN	113 e	27 e	90 e	1.91 e
40% MN + PGPR	95 f	22 f	76 f	2.26 e
20% MN	54 g	13 g	45 g	0.81 g
20% MN + PGPR	56 g	10 h	42 g	1.12 f

There is no significant difference between means with the same letter in the same column (*p* < 0.05).

*MF* Mineral fertilizer, *PGPR* Plant growth promoting rhizobacteria.

The present study underscores the multifaceted role of PGPR in influencing both macro and micronutrient dynamics in lettuce plants, shedding light on potential effectiveness across different nutrient categories and under varying levels of mineral fertilizer application. Applying PGPR with reduced mineral fertilizer can assist plants absorb more nutrients by optimizing the distribution of available nutrients to plants^29,44^. Wang et al.^45^ reported the existence of nitrogenase which plays a crucial role in the biological nitrogen fixation process. Certain PGPR are classified as phosphate-solubilizing bacteria, and many bacteria, including various strains of *Bacillus*, can convert insoluble phosphorus into soluble forms^11,45^. Availability in the rhizosphere can also be explained by plants' increased mineral ion uptake through PGPR stimulation of the proton pump ATPase. This effect can also be attributed to the production of organic acids in the rhizosphere by both plants and bacteria, which lower soil pH and increase the availability of elements^46–48^. Several studies reported that beneficial bacteria significantly increased N, P, K, Ca^40,44,49,50^, Mg, Fe, Mn, Zn and Cu^44,50^ concentrations of the lettuce compared with the control.

It is possible that the higher nitrate concentration in the PGPR-treated lettuces was due to the fixation of atmospheric nitrogen by PGPR. Kaymak et al.^49^ reported similar results that the bacterial mixture of *Pseudomonas putida*,* Pseudomonas fluorescens*, and *Bacillus megaterium* increased the nitrate content of lettuce leaves. In our study, the bacteria mixture contained *Bacillus subtilis*, *Bacillus megaterium*, and *Pseudomonas fluorescens.* However, contrary to our results, in some studies, PGPR applications reduced nitrate content in lettuce leaves^22,31^. Nitrate accumulation in lettuce can vary significantly depending on lettuce type and cultivation conditions. Studies have shown that nitrate levels in curled lettuce ranged from 16 to 3400 mg kg^−1^ FW (average of 1601 mg kg^−1^ FW from 301 samples)^51^. In our study, nitrate concentrations were well below the harmful limits for human health. The commercialization threshold is set at 5000 mg kg^−1^ FW, the maximum level imposed by the European Commission (EC Reg. No. 1258/2011) for protected-grown lettuce grown under cover from October to March, as is our case^51^.

The inoculation of PGPR has resulted in heightened levels of total phenolic and flavonoid contents and increased TSS and EC in lettuce leaves (Fig. 4)^21,52^. PGPR increased total phenolic and flavonoid productions when mineral fertilizer was reduced. This phenomenon may suggest that the synergy between reduced mineral fertilizer application and PGPR utilization contributes to an augmentation in the production of phenolic compounds, indicative of a potential synergistic effect on secondary metabolite synthesis. This effect aligns with previous findings that PGPR can enhance antioxidant compounds in hydroponically cultivated basil^9^ and spinach^19^, including total phenols, flavonoids, vitamin C, and TSS. Moreover, the lettuce leaves grown with up to a 60% reduction in mineral fertilizer, as explored in this study, exhibited mineral element concentrations—including potassium, calcium, magnesium, iron, and zinc (Tables 3, 4). Theseare consistent with the reported mineral content in lettuce by Kim et al.^53^. The cultivation under reduced MF conditions, combined with PGPR inoculation, thus positively influenced the secondary metabolites and the essential mineral composition of lettuce leaves.

While the specific results are not presented in this study, it is noteworthy that PGPR demonstrates potential as a biocontrol agent, contributing to the cultivation of healthy lettuce by impeding the development of harmful bacteria and other microorganisms in the root environment^11,54^. This observed biocontrol capability suggests a potential alternative to using pesticides during cultivation, highlighting the prospect of cultivating lettuce without the need for chemical pesticides. This shift toward a pesticide-free approach holds significant importance in terms of agricultural sustainability and human nutrition, aligning with the broader goals of promoting healthier and environmentally friendly cultivation practices.

In conjunction with this study's exploration, it is essential to acknowledge some limitations. One limitation inherent in this study and in others of similar studies pertain to the understanding of underlying mechanisms. Despite advancements in comprehending how PGPR contribute to plant growth, there remains a substantial knowledge gap. The understand the nature of plant–microbe interactions across diverse environments necessitates further investigation to elucidate the intricate processes at play. The primary objective of this study was to showcase the practical applicability of PGPRs as biofertilizers in hydroponic farming, concentrating on agronomic and quality analyses rather than delving into the physiological mechanisms of PGPRs. Noteworthy distinctions were identified and demonstrated in the above. However, it is crucial to acknowledge that a more comprehensive examination of factors such as organic acids, phytohormones secreted by bacteria, and bacterial charge in the culture media post-harvest would enhance the interpretation of results from a physiological standpoint^46,48,55^.

Moreover, the use of PGPR in plant nutrition has shown promise^56^, but several constraints and challenges are associated with their application. Some of these limitations are^11,55^: (1) Strain specificity: Certain strains may benefit certain plants or under certain environmental conditions. Plant responses to PGPR can vary depending on plant species, developmental stage and specific physiological conditions, (2) Environmental factors: The efficacy of PGPR is often influenced by environmental factors such as root zone pH, temperature, organic matter, other microorganisms and chemical substances such as fertilizers or pesticides. Inconsistent environmental conditions can affect the ability of PGPR to function optimally, (3) Competition with other micro-organisms beneficial or not beneficial: Other microorganisms in the root zone can outcompete introduced PGPR strains, reducing their colonization and persistence in the rhizosphere. This competition may limit the long-term benefits of PGPR, (4) Regulatory approval and standardization: There is a need for standardized formulations and regulatory frameworks for PGPR products. Finally, as presented above the complexity of plant–microbe interactions in different environments requires further research to unravel the underlying mechanism processes.

## Conclusion

In hydroponic farming, mineral fertilizers can be reduced by employing PGPR. According to the findings of this study, mineral fertilizers can be reduced by 20% to 40%. Although this may lead to a slight decrease in yield, it results in an improved mineral nutritional and product quality of lettuce. The potential benefits of reduced mineral fertilizers, financial savings, environmental conservation, and enhanced nutritional and antioxidant contents in lettuce, make it a promising approach in hydroponic cultivation. Resolving the limitations mentioned above for PGPR could facilitate more rapid and widespread adoption of these beneficial microorganisms in soilless cultivation. In future research efforts, it is suggested:

- Detailed examination of bacterial colonization in SCS: A more thorough investigation into the colonization dynamics of bacteria in SCS and water culture could provide valuable insights, e.g.the frequency with which PGPR should be introduced into the root medium for optimal effects.
- Exploration of new bacterial strains and optimum doses for SCS: Research could focus on identifying novel bacterial strains specifically tailored for SCS and determining the optimum doses for practical application.
- Study on the shelf life of PGPR applied to lettuces: A dedicated examination into the shelf life of PGPR applied to lettuces can offer practical insights into the longevity of the beneficial effects by better understanding the temporal dynamics of PGPR.
- Investigation into the suppression of root diseases: Future studies can delve into the potential of PGPR to suppress root diseases in SCS. This research could address a critical aspect of plant health and contribute to further developing sustainable disease management strategies.

## Data Availability

The data presented in this study are available in the article.

## Abbreviations

ACC: 1-Aminocyclopropane-1-carboxylic acid
ANOVA: Analysis of variance
ATP: Adenosine triphosphate
Ca: Calcium
Cu: Copper
DW: Dry weight
E: East
EC: Electrical conductivity
EC: European commission
Fe: Iron
FW: Fresh weight
HCI: Hydrochloric acid
IAA: Indole-3-acetic acid
K: Potassium
MF: Mineral fertilizer
Mg: Magnesium
Mn: Manganese
N: Nitrogen
N: North
NO_3_: Nitrate
P: Phosphorus
PGPR: Plant growth promoting rhizobacteria
SCS: Soilless culture systems
SPAD: Soil plant analysis development
TA: Titratable acidity
TSS: Total soluble solids
UV: Ultraviolet
Zn: Zinc
®: Registered trademark symbol
°C: Degree celsius
